# Establishment and validation of survival nomogram score staging for esophageal squamous cell carcinoma patients after minimally invasive surgery combined with immune prognostic index and clinicopathological features

**DOI:** 10.3389/fimmu.2025.1635000

**Published:** 2025-09-17

**Authors:** Shao-jun Xu, Cheng-xiong You, Zhao-min Sun, Chao Chen, Yan-ming Shen, Jin Huang, Yun-fan Luo, Jie Chen, Ji-hong Lin, Shu-chen Chen

**Affiliations:** ^1^ Department of Thoracic Surgery, Fujian Medical University Union Hospital, Fuzhou, Fujian, China; ^2^ Key Laboratory of Ministry of Education for Gastrointestinal Cancer, Fujian Medical University, Fuzhou, Fujian, China; ^3^ Key Laboratory of Cardio-Thoracic Surgery, Fujian Medical University, Fujian Province University, Fuzhou, China

**Keywords:** esophageal squamous cell carcinoma, minimally invasive esophagectomy, immune prognostic index, nomogram, survival nomogram-scoring staging

## Abstract

**Background:**

Minimally invasive esophagectomy (MIE) is commonly applied in the treatment of esophageal squamous cell carcinoma (ESCC). The preoperative immune prognostic index (IPI) is considered a potential indicator for ESCC. In this study, we attempted to establish and validate a comprehensive clinical risk staging toward predicting the long-term outcome in ESCC patients after MIE through the combined application of IPI and clinicopathological features based on nomogram.

**Methods:**

Patients diagnosed with ESCC and who were treated with MIE at our center were randomly assigned in a 7:3 ratio to two cohorts: training (n=425) and validation (n=188). Nomograms were then established according to the Cox multivariate results for the training cohort. To evaluate the predictive value and the clinical benefit rate of the nomogram, we employed the calibration curve, decision curve analysis (DCA), and time-dependent area under the curve (t-AUC). The best cut-off value obtained was applied to develop the survival nomogram-scoring (SNS) staging.

**Results:**

Patients with ESCC and who underwent MIE exhibited the worse overall survival (OS) and disease-free survival (DFS) with an IPI=2 relative to IPI=0 or 1 (all P < 0.001) in the training and validation cohorts, respectively. According to the multivariate Cox analysis, we established nomograms that combined IPI with the clinicopathological features to predict the OS and DFS. The t-AUC and DCA demonstrated that the nomograms had a better predictive value and clinical benefit rate than the American Joint Committee on Cancer (AJCC) staging. Accordingly, we developed the SNS staging and found that the OS and DFS were significantly different among patients at different stages (all P < 0.001). The dynamic mortality risk rate revealed a double peak in the high-risk patients (12 and 48 months) and a single peak in the low–risk patients (24 months) and middle-risk patients (12 months), which gradually decreased over time.

**Conclusion:**

The SNS staging based on IPI and clinicopathological features can thus be applied to effectively evaluate the OS and DFS of ESCC patients after MIE. Our findings may facilitate the development of clinical follow-up strategies.

## Introduction

Esophageal squamous cell carcinoma (ESCC) is one of the most common types of esophageal cancer in Asia, accounting for approximately 90% of all cases (1). Minimally invasive esophagectomy (MIE) is less invasive and has a better prognosis than open esophagectomy (OE), which makes it the most important surgical method in the treatment of ESCC (2, 3). Presently, no tool can effectively predict the prognosis of patients with ESCC. Therefore, it is necessary to develop a prediction model that allows the measurement of the long-term oncology outcome on patients.

Recent evidence indicates that, in addition to tumor characteristics, immune-inflammatory responses in the tumor microenvironment play a critical role in promoting tumorigenesis and progression (4, 5). Past studies have demonstrated that blood indicators, such as derived neutrophil/lymphocyte ratio (dNLR), lymphocyte/monocyte ratio (LMR), systemic immune-inflammation index (SII), and serum lactate dehydrogenase (LDH), can help measure the degree of immune inflammatory response in the tumor microenvironment, which has significant potential for predicting the disease progression in patients with a variety of cancers (6–9). The immune prognostic index (IPI) established by combining dNLR and LDH is considered to be a better biomarker for reflecting the degree of immune inflammatory response in the body than a single index and has demonstrated excellent predictive performance in the lung, esophageal and gastric cancers (10–12).

Nomogram is a tool that can individually and visually evaluate the clinical prognosis in cancer patients, showing accurate predictive value (13–15). Therefore, the present work attempts to establish a new clinical staging system, namely the survival nomogram scoring staging (SNS) that comprehensively considers tumor characteristics (T and N stage) and tumor immunoinflammatory response (IPI) to predict the long-term oncology outcomes in ESCC after MIE and validates its risk prediction values in an independent cohort.

## Materials and methods

### Study population

We retrospectively analyzed the data of 613 ESCC patients at our institution and randomly assigned the patients (7:3 ratio) to training (n=425) or validation (n=188) cohorts. The inclusion criteria were as follows: (1) pathological diagnosis of ESCC after MIE; (2) radical resection with a negative margin (R0); (3) primary tumor did not metastasize or invade the surrounding tissues and organs; (4) no preoperative neoadjuvant therapy was performed. The exclusion criteria were as follows: (1) patients who received treatment with OE; (2) patients who died within a short period after the surgery (within 30 days); (3) lack of clinicopathological information; (4) follow-up time being <3 months.

The Ethics Review Committee of the Fujian Medical University Union Hospital approved our study protocol.

### Clinical data collection and definition

The perioperative information that was assessed included the following: sex, age, body mass index (BMI), ASA score, surgical procedure, tumor location, lymphadenectomy, intraoperative bleeding, and postoperative adjuvant chemotherapy. In addition, we collected common pathological features such as the T stage, N stage, and histologic grade for analyses.

Most patients underwent McKeown–MIE and some underwent Ivor Lewis–MIE. The methods of lymphadenectomy included either two-field or three-field lymph node dissection.

Blood samples were collected from all patients within 7 days before surgery for routine blood examinations and biochemical tests, and the data of neutrophil count, white blood cell count, and LDH concentration were collected. The dNLR was calculated based on past reports by Proctor et al. (16). The IPI scores were calculated from dNLR (< 1.88 was defined as 0; ≥1.88 was defined as 1 score) and LDH (<190.5 IU/L was defined as 0 scores; ≥190.5 IU/L was defined as 1, with reference to a past study (12).

### Follow-up of patients

The patients were followed up every 3 months for 1 year and 6 months until 5 years after the surgery, and then annually for >5 years until the patient’s death or the final follow-up cutoff in December 2019. Most routine follow-ups included physical examination, laboratory tests, chest computed tomography, abdominal and pelvic ultrasound, general examination if necessary, and annual digestive endoscopy. The interval time between radical surgery and death from any cause or the final follow-up was defined as the overall survival (OS), and the time between the surgery and tumor progression, recurrence, or death was defined as the disease-free survival (DFS).

### Statistical analysis

The included ESCC patients were randomly assigned to 2 groups (training and validation cohorts; 7:3 ratio). Continuous variables were analyzed by Mann–Whitney U-test. The Pearson’s Chi-squared test or Fisher’s exact test was employed to distinguish categorical variables between the different groups. Kaplan–Meier and log-rank tests were employed to analyze the OS and DFS among the different groups. Cox proportional hazards multivariate analysis was employed to determine the independent influencing factors for ESCC patients, and a nomogram combining IPI and clinicopathological features was established based on the results. The calibration curve of the bootstrap 1000 resampling method was applied to test the prediction ability of the nomogram. The time-dependent area under the curve (t-AUC) was employed to compare the predictive performance of the two nomograms and the American Joint Committee on Cancer (AJCC) staging. In addition, we applied the true and false positive rates of different risk thresholds analyzed by decision curve analysis (DCA) to measure the clinical benefit rate of the two nomograms and the AJCC staging (17). The total score was calculated for each patient based on the score of the variables included in the nomogram. We also used the X-tile software (version 3.6.1) to determine the best cutoff values for predicting OS and DFS, and the entire cohort was classified into 3 groups (18). Finally, the hazard assessment function analyzes the risk information of different groups across different periods.

P < 0.05 was defined as statistically significant and the tests in the study were two-sided. Statistical methods in the study were analyzed using SPSS 23.0 and R language 3.6.3.

## Results

### Comparison of clinicopathological information between two cohorts

**Table 1** shows the clinicopathological information of the two cohorts. In the training cohort, there were 425 ESCC patients, of which 139 (32.7%) patients were women and 286 (67.3%) were men. There were 331 (77.9%) patients aged ≤65 years while 94 (22.1%) were aged > 65 years. Among the enrolled patients, the IPI value was 0 in 214 (50.4%), 1 in 174 (40.9%), and 2 in 37 (8.7%) patients.

**Table 1:** Compare the clinicopathological characteristics of patients in training cohort with validation cohort.

Characteristics	Training cohort[n (%)]	Validation cohort[n (%)]	*P* value
	(n=425)	(n=188)	
Sex			0.478
Female	139(32.7)	67(35.6)	
Male	286(67.3)	121(64.4)	
Age			0.150
≤65	331(77.9)	156(83.0)	
>65	94(22.1)	32(17.0)	
BMI (kg/m2)			0.495
≤18.5	41(9.6)	24(12.8)	
18.5-25	322(75.8)	139(73.9)	
≥25	62(14.6)	25(13.3)	
ASA score			0.821
I/II	358(84.2)	157(83.5)	
III/IV	67(15.8)	31(16.5)	
Histologic grade			0.277
Gx/G1	184(43.3)	72(38.3)	
G2	192(45.2)	98(52.1)	
G3	49(11.5)	18(9.6)	
Tumor location			0.236
Proximal	44(10.4)	12(6.4)	
Mid	267(62.8)	128(68.1)	
Distal	114(26.8)	48(25.5)	
T stage			0.743
T1	119(28.0)	47(25.0)	
T2	78(18.4)	36(19.1)	
T3/T4a	228(53.6)	105(55.9)	
N stage			0.159
N0	170(53.3)	131(54.1)	
N1	85(26.6)	55(22.7)	
N2/3	64(20.1)	56(23.1)	
TNM stage			0.422
I	119(28.0)	48(25.5)	
II	129(30.4)	51(27.1)	
III/IVA	177(41.6)	89(47.3)	
Lymphadenectomy			0.906
Two-field	383(90.1)	170(90.4)	
Three-field	42(9.9)	18(9.6)	
Surgical procedure			0.558
McKeown	380(89.4)	171(91.0)	
Ivor Lewis	45(10.6)	17(9.0)	
Intraoperative bleeding(ml)			0.074
≤100	214(50.4)	104(55.3)	
100-200	158(37.2)	53(28.2)	
≥200	53(12.5)	31(16.5)	
IPI Score			0.452
IPI=0	214(50.4)	105(55.9)	
IPI=1	174(40.9)	68(36.2)	
IPI=2	37(8.7)	15(8.0)	
Adjuvant chemotherapy			0.106
No	220(51.8)	84(44.7)	
Yes	205(48.2)	104(55.3)	

Of the 188 patients in the validation cohort, 67 (35.6%) were women and 121 (64.4%) were men. 156 (83.0%) patients were ≤65 years while 32 (17.0%) patients were > 65 years. The IPI value was 0 in 105 (55.9%), 1 in 68 (36.2%), and 2 in 15 (8.0%) patients. There were no differences in sex, age, IPI score, TNM stage, BMI, ASA score, tumor location, histologic grade, surgical procedure, T stage, N stage, intraoperative bleeding, and postoperative adjuvant chemotherapy between the two cohorts (P > 0.05, **Table 1**).

### Survival analysis in two cohorts

**Figure 1:**
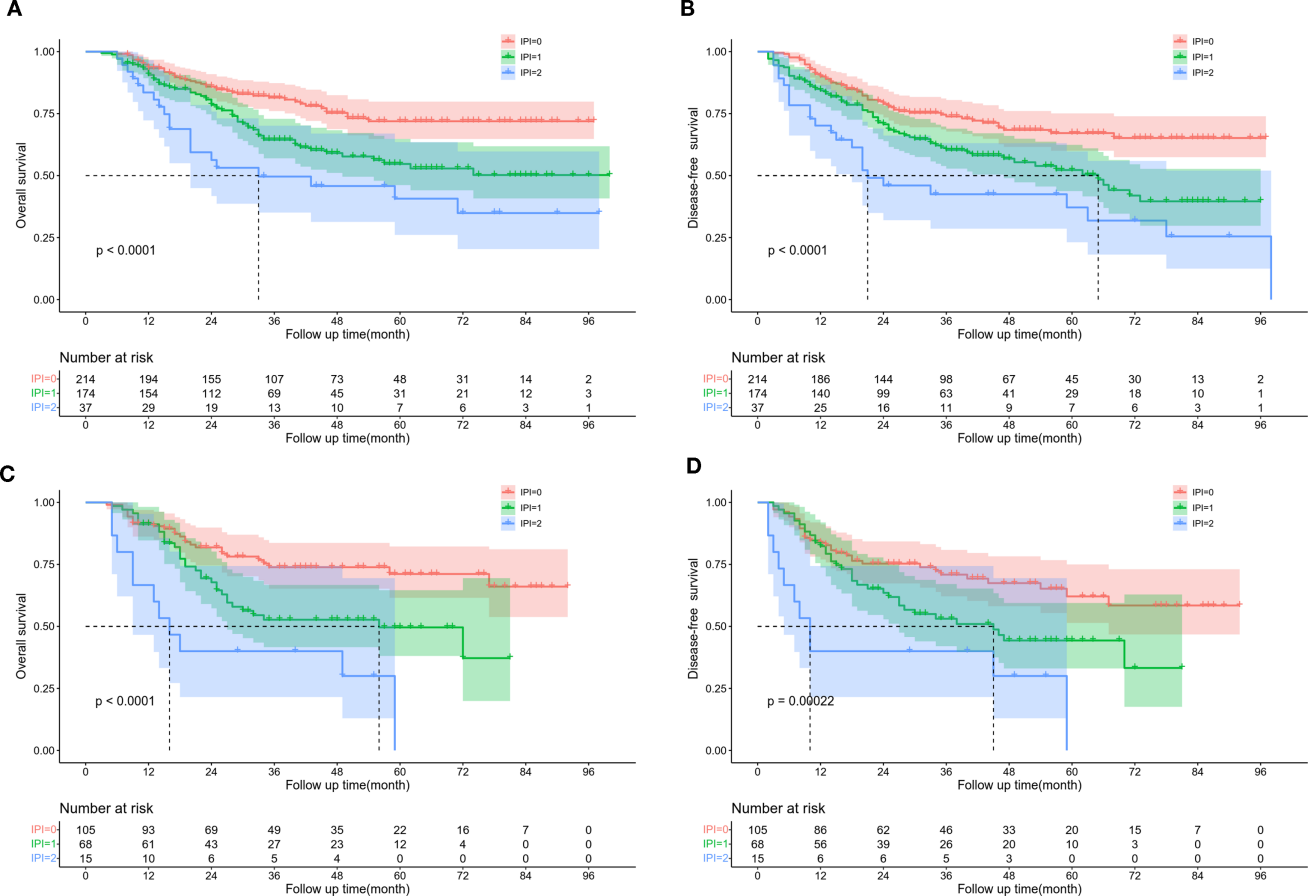
Kaplan Meier survival curve of ESCC patients according to IPI score. **(A)** OS and **(B)** DFS in training cohort. **(C)** OS and **(D)** DFS in validation cohort.

We analyzed the effects of dNLR, LDH, and IPI on the OS in the training cohort and found that the 5-year OS rates of high dNLR and low dNLR were significantly different (67.3% VS 48.4%, P < 0.001, **Supplementary Figure S1A**). The 5-year OS rates of patients in the high LDH and low LDH groups were also significant differences (66.4% VS 51.8%, P=0.007, **Supplementary Figure S1C**). The 5-year OS rate of patients in IPI=0, IPI=1, and IPI=2 was 71.9%, 54.7%, and 40.7%, respectively (P < 0.001, **Figure 1A**). Moreover, the 5-year DFS rate of patients in the low dNLR group was significantly shorter than that in the high dNLR group (46.2% VS 63.2%, P < 0.001, **Supplementary Figure S1B**). The 5-year DFS rate of patients with high LDH was significantly longer than those of patients with low LDH (62.5% VS 48.4%, P=0.0066, **Supplementary Figure S1D**). The 5-year DFS rate of patients with IPI=0, IPI=1, and IPI=2 was 67.1%, 52.3%, and 37.2%, respectively (P < 0.001, **Figure 1B**).

In the validation cohort, the 5-year OS rate of patients in the high and low dNLR groups were 65.1% and 42.4%, respectively (P=0.017, **Supplementary Figure S2A**). The 5-year OS rate of patients in the high LDH and low LDH groups were 65.8% and 30.5%, respectively (P < 0.001, **Supplementary Figure S2C**). The 5-year OS rate of patients in IPI=0, IPI=1, and IPI=2 was 71.1%, 49.6%, and 0%, respectively (P < 0.001, **Figure 1C**). The 5-year DFS rate of patients in the high and low dNLR groups were 57.4% and 41.5%, respectively (P=0.018, **Supplementary Figure S2B**). The 5-year DFS rate of patients in the high and low LDH groups were 57.3% and 25.8%, respectively (P=0.0022, **Supplementary Figure S2D**). The 5-year DFS of patients with IPI=0, IPI=1, and IPI=2 was 62.1%, 44.3%, and 0%, respectively (P < 0.001, **Figure 1D**). The dNLR, LDH, and IPI were the risk factors identified for the prognosis of ESCC in the 2 groups.

Furthermore, we used the t-AUC to compare the performance of preoperative dNLR, LDH, and IPI in the training cohort. The AUC of preoperative dNLR, LDH, and IPI for predicting the 5-year OS rate was 0.554 (95%CI, 0.488–0.621), 0.569 (95%CI, 0.505–0.633), and 0.598 (95%CI, 0.526–0.671), respectively (**Supplementary Figure S3A**). The AUC of 5-year DFS rate was 0.535 (95%CI, 0.469–0.600), 0.567 (95%CI, 0.504–0.629), and 0.581 (95%CI, 0.509–0.653), respectively (**Supplementary Figure S3B**). The predictive performance of preoperative IPI in predicting the 5-year OS rate and the DFS rate was greater than those of dNLR and LDH. Similarly, the AUC of preoperative IPI for predicting the 5-year OS (**Supplementary Figure S3C**) and DFS (**Supplementary Figure S3D**) was the largest in the validation cohort.

### Effect of preoperative IPI on the prognosis of ESCC patients

In univariate analysis, IPI score, age, ASA score, T stage, N stage, and intraoperative bleeding were identified as the influencing risk factors of the OS in ESCC patients after MIE (all P < 0.05). Multivariate Cox analysis indicated that the IPI score (IPI=2 vs IPI=0: HR, 2.776; 95% CI, 1.598–4.824; P < 0.001; IPI=1 vs IPI=0: HR, 1.849; 95% CI, 1.247–2.744; P=0.002), age (>65 years vs ≤65 years: HR, 1.610; 95% CI, 1.093–2.373; P=0.016), T stage (T3/T4a vs T1: HR, 3.483; 95% CI, 1.773–6.843; P < 0.001; T2 vs T1: HR, 2.216; 95% CI, 1.034–4.750; P=0.041), N stage (N2/3 vs N0: HR, 2.662; 95% CI, 1.700–4.169; P < 0.001; N1 vs N0: HR, 2.229; 95% CI, 1.410–3.525; P=0.001), and intraoperative bleeding (≥200 vs ≤100: HR, 2.649; 95% CI, 1.491–4.709; P=0.001) were the independent influencing factors for poor OS (**Table 2**).

**Table 2:** Univariate and multivariate Cox analysis for OS and DFS in the training cohort.

Characteristics	Overall survival				Disease-free survival			
	Univariate analysis		Multivariate analysis		Univariate analysis		Multivariate analysis	
	HR (95%CI)	*P*-value	HR (95%CI)	*P*-value	HR (95%CI)	*P*-value	HR (95%CI)	*P*-value
Sex								
Female								
Male	1.219(0.830-1.791)	0.312			0.943(0.655-1.359)	0.754		
Age(year)								
≤65								
>65	1.804(1.233-2.641)	0.002	1.610(1.093-2.373)	0.016	1.714(1.206-2.435)	0.003	1.535(1.074-2.193)	0.019
BMI (kg/m2)								
≤18.5								
18.5-25	0.804(0.472-1.368)	0.42			0.993(0.587-1.678)	0.978		
≥25	0.918(0.476-1.773)	0.799			1.238(0.664-2.310)	0.502		
ASA score								
I/II								
III/IV	1.652(1.029-2.651)	0.038	1.400(0.860-2.280)	0.176	1.520(0.979-2.361)	0.062		
Histologic grade								
Gx/G1								
G2	0.952(0.656-1.382)	0.797			0.910(0.650-1.274)	0.582		
G3	1.269(0.728-2.213)	0.401			1.043(0.614-1.773)	0.876		
Tumor location								
Proximal								
Mid	1.011(0.562-1.818)	0.97			1.149(0.656-2.011)	0.628		
Distal	1.087(0.575-2.055)	0.797			1.151(0.628-2.112)	0.649		
T stage								
T1								
T2	3.105(1.462-6.594)	0.003	2.216(1.034-4.750)	0.041	2.410(1.333-4.358)	0.004	1.712(0.938-3.127)	0.08
T3/4a	5.664(2.953-10.866)	<0.001	3.483(1.773-6.843)	<0.001	3.614(2.193-5.957)	<0.001	2.341(1.383-3.964)	0.002
N astage								
N0								
N1	2.566(1.650-3.991)	<0.001	2.229(1.410-3.525)	0.001	2.096(1.404-3.129)	<0.001	1.890(1.247-2.865)	0.003
N2/3	3.453(2.275-5.240)	<0.001	2.662(1.700-4.169)	<0.001	3.131(2.156-4.546)	<0.001	2.565(1.714-3.838)	<0.001
Lymphadenectomy								
Two-field								
Three-field	0.812(0.456-1.444)	0.478			0.815(0.477-1.393)	0.815		
Surgical procedure								
McKeown								
Ivor Lewis	0.725(0.408-1.289)	0.273			0.861(0.526-1.410)	0.551		
Intraoperative bleeding(ml)								
≤100								
100-200	1.223(0.842-1.776)	0.291	1.300(0.892-1.895)	0.172	1.173(0.833-1.651)	0.36	1.268(0.899-1.789)	0.176
≥200	2.081(1.191-3.636)	0.01	2.649(1.491-4.709)	0.001	1.962(1.190-3.236)	0.008	2.450(1.469-4.087)	0.001
IPI Score								
IPI=0								
IPI=1	1.879(1.280-2.758)	0.001	1.849(1.247-2.744)	0.002	1.717(1.215-2.427)	0.002	1.729(1.216-2.457)	0.002
IPI=2	3.157(1.863-5.349)	<0.001	2.776(1.598-4.824)	<0.001	2.901(1.788-4.705)	<0.001	2.652(1.614-4.355)	<0.001
Adjuvant chemotherapy								
No								
Yes	1.342(0.946-1.906)	0.1			1.313(0.955-1.806)	0.094		

Similarly, age, T stage, N stage, intraoperative bleeding, and IPI score were identified as the influencing factors of DFS in ESCC patients (all P < 0.05). Multivariate analysis revealed that the IPI score (IPI=2 vs IPI=0: HR, 2.652; 95% CI, 1.614–4.355; P < 0.001; IPI=1 vs IPI=0: HR, 1.729; 95% CI, 1.216–2.457; P=0.002), age (>65 years vs ≤65 years: HR, 1.535; 95% CI, 1.074–2.193; P=0.019), T stage (T3/T4a vs T1: HR, 2.341; 95% CI, 1.383–3.964; P=0.002; T2 vs T1: HR, 1.712; 95% CI, 0.938–3.127; P=0.08), N stage (N2/3 vs N0: HR, 2.565; 95% CI, 1.714–3.838; P < 0.001; N1 vs N0: HR, 1.890; 95% CI, 1.247–2.865; P=0.003), and intraoperative bleeding (≥200 vs ≤100: HR, 2.450; 95% CI, 1.469–4.087; P=0.001) were identified as the independent risk factors affecting the DFS (**Table 2**).

### Establishment and validation of a survival nomogram combining IPI and clinicopathological features

**Figure 2:**
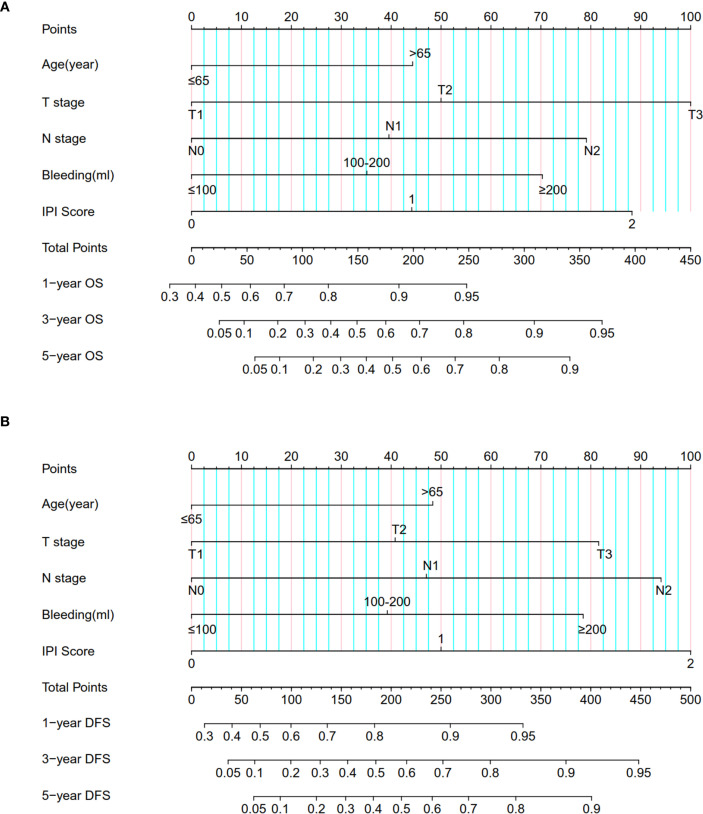
Nomogram for predicting **(A)** OS and **(B)** DFS in ESCC patients by combining IPI score and clinicopathological information.

Based on the results of Cox multivariate analyses, we established survival nomograms by combining IPI score and clinicopathological features (**Figures 2A, B**). The predictive calibration curves of the nomogram in predicting 1-, 3-, and 5-year OS (**Supplementary Figures S4A, C, E**) and DFS (**Supplementary Figures S4B, D, F**) were in good agreement with the actual results of the training cohort. Furthermore, the prediction results of the nomogram were relatively consistent with the actual observation results in the validation cohort (**Supplementary Figures S5A–F**).

### Comparison of predictive power and clinical value of the survival nomogram and the 8^th^ AJCC staging

**Figure 3:**
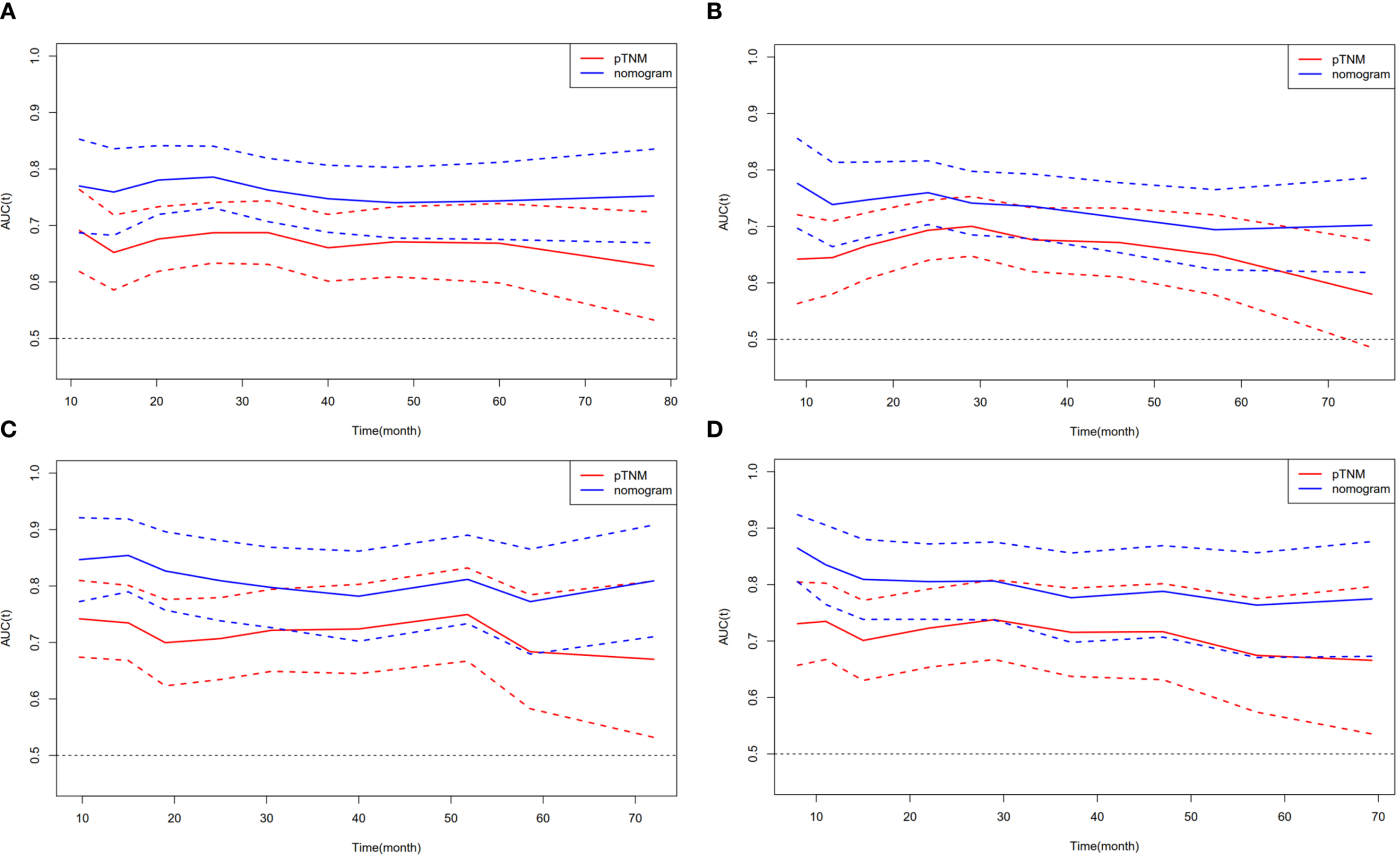
Compare the predictive ability of the nomogram with the 8^th^ AJCC staging. t-AUC for **(A)** OS and **(B)** DFS in training cohort. **(C)** t-AUC for OS and **(D)** DFS in validation cohort.

We further compared the predictive ability of the survival nomogram and the 8^th^ AJCC staging to predict the OS and DFS of ESCC patients after MIE. The AUC of the survival nomogram in the training cohort was greater than that of the 8^th^ AJCC staging. The t-AUC range of the 8^th^ AJCC staging prediction of the OS of ESCC patients was 0.676–0.687, while the survival nomogram was 0.740–0.780 (**Figure 3A**). The t-AUC range of DFS predicted by the 8^th^ of the AJCC staging and survival nomogram was 0.666–0.700 and 0.715–0.747, respectively (**Figure 3B**). Similarly, in the validation group, we found that the t-AUC range of the 8^th^ AJCC staging was 0.700–0.750 and the survival nomogram range was 0.786–0.827 (**Figure 3C**). The t-AUC range of the DFS predicted by the 8^th^ AJCC staging and the survival nomogram was 0.701–0.717 and 0.788–0.809, respectively (**Figure 3D**).

**Figure 4:**
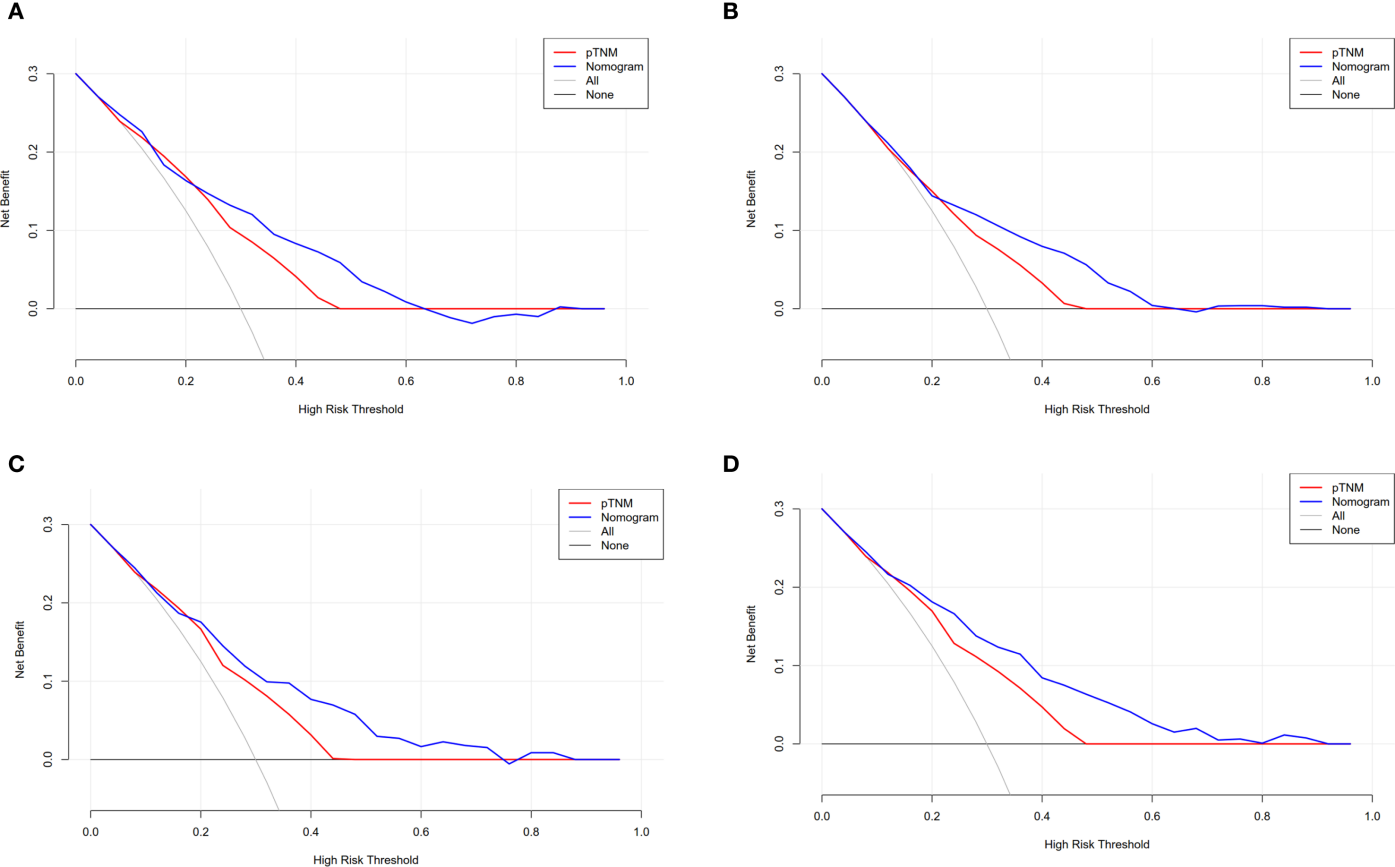
Compare the clinical application value of the nomogram with the 8^th^ AJCC staging. DCA for **(A)** OS and **(B)** DFS in training cohort. **(C)** DCA for OS and **(D)** DFS in validation cohort.

We used the DCA to analyze the clinical benefits of the 8^th^ AJCC staging and survival nomogram. Within a certain range, the survival nomogram for predicting OS (0.32–0.62) (**Figure 4A**) and DFS (0.22–0.60) (**Figure 4B**) in the training cohort was better than that in the 8^th^ AJCC staging. Similarly, the survival nomogram predicting OS (0.18–0.78) (**Figure 4C**) and DFS (0.16–0.80) (**Figure 4D**) were more clinically useful than the 8^th^ AJCC staging for a considerable range of the validation cohort.

### A survival nomogram scoring staging was developed to stratify the patients at risk

**Figure 5:**
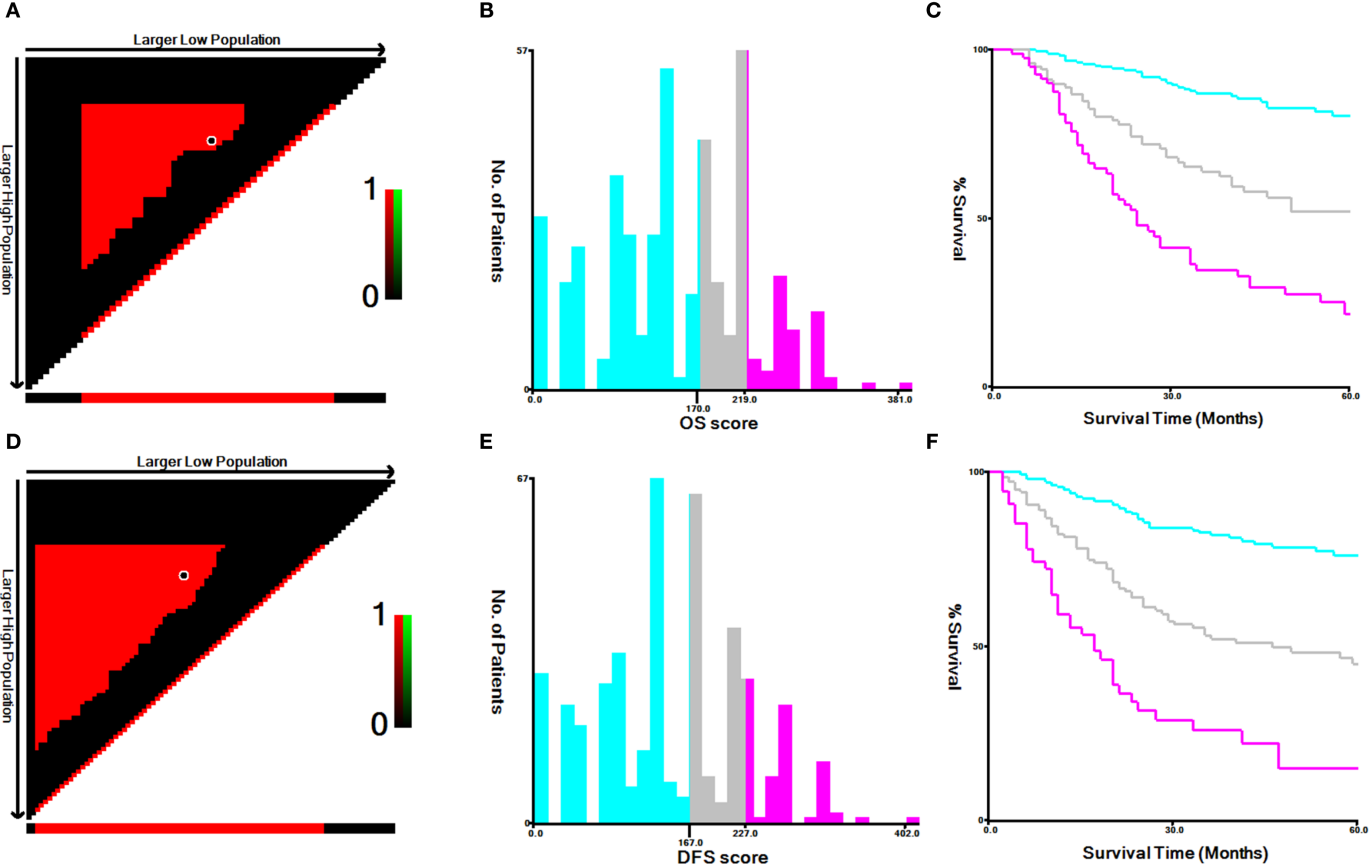
The best cut-off values for **(A–C)** OS and **(D–F)** DFS according to X-tile in training cohort.

**Figure 6:**
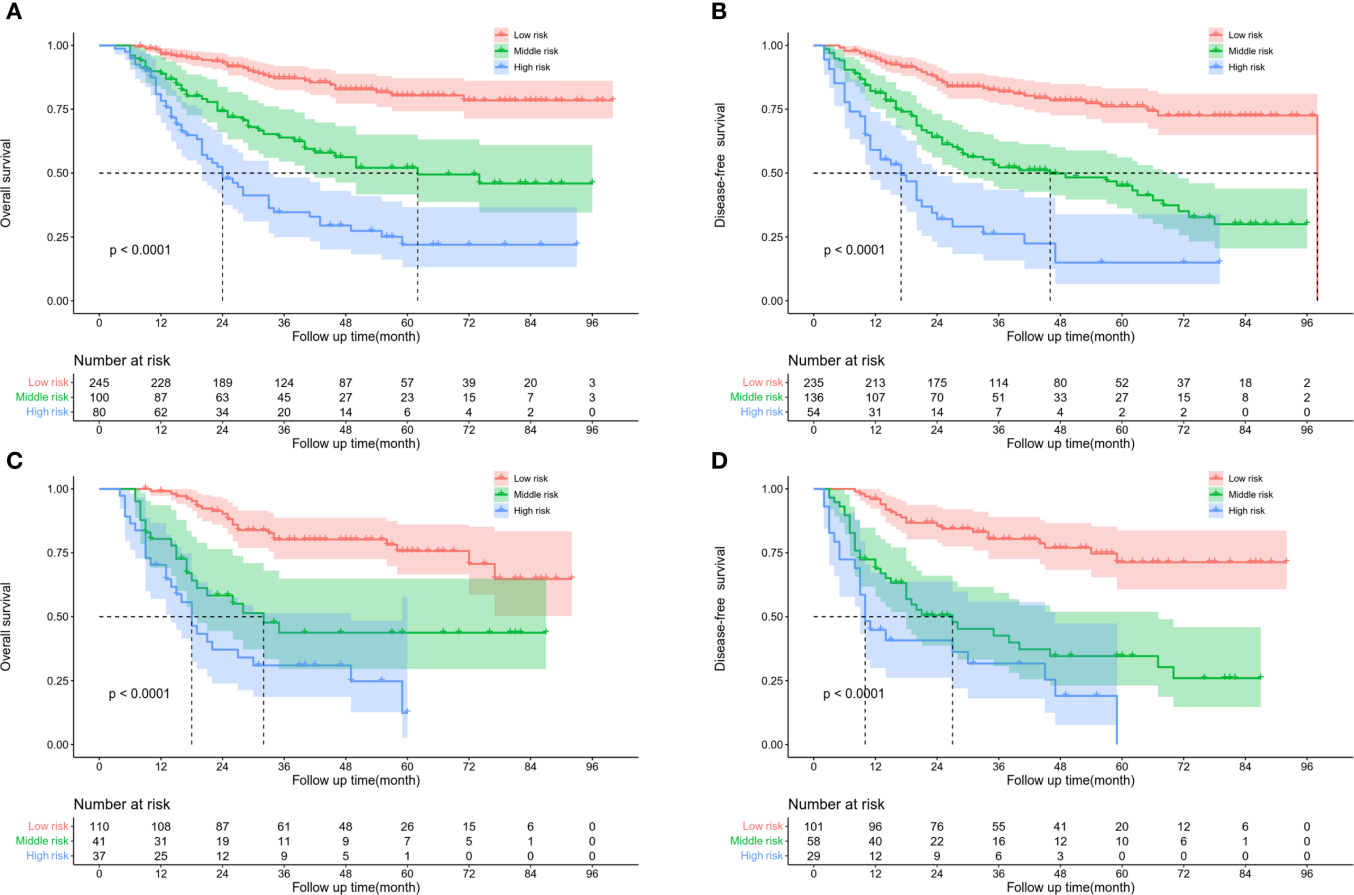
Kaplan Meier survival curve of ESCC patients according to SNS staging. **(A)** OS and **(B)** DFS in training cohort. **(C)** OS and **(D)** DFS in validation cohort.

By using the X-tile software, the best cut-off values of OS (**Figures 5A–C**) and DFS (**Figures 5D–F**) were stratified to develop the survival nomogram score (SNS) staging. The patients were accordingly classified into 3 groups based on the OS scores in the training cohort: low risk (≤170 score), middle risk (170–219 score), and high risk (>219 score). The patients were also classified as low risk (≤167 score), middle risk (167–227 score), and high risk (>227 score) based on their DFS score. The 5-year OS of patients in the low-risk, middle-risk, and high-risk groups were 80.4%, 52.1%, and 21.9%, respectively (**Figure 6A**). The 5-year DFS were 76.1%, 45.1%, and 15.0%, respectively (**Figure 6B**). In the validation cohort, there were significant differences in the OS of the low risk (≤170 score), middle risk (170–219 score), and high risk (>219 score) groups, with the 5-year OS of 75.7%, 43.7%, and 12.4%, respectively (**Figure 6C**). In addition, the DFS of patients in the low risk (≤167 score), middle risk (167–227 points), and high risk (>227 points) were significantly different, with the 5-year DFS of 71.3%, 34.6%, and 0%, respectively (**Figure 6D**).

### Dynamic mortality risk rate assessment by SNS staging

**Figure 7:**
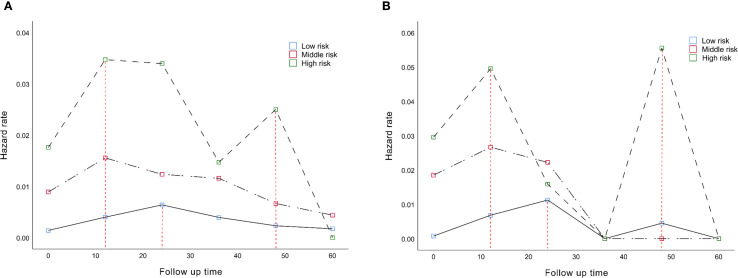
Dynamic mortality risk of different SNS staging in **(A)** training cohort and **(B)** in validation cohort.

We analyzed changes in the hazard rate (HR) which indicates the rate of death over time, by measuring SNS over a 5-year follow-up period. In the training cohort, patients with low and moderate risk had the highest probability of death at 24 (peak HR=0.0064) and 12 months (peak HR=0.011), respectively. However, the probability of death in the high-risk patients showed two peaks, at 12 (peak HR=0.034) and 48 months (peak HR=0.025)(**Figure 7A**). Similarly, in the validation cohort, the highest probability of death was estimated at 24 (peak HR=0.011) and 12 months (peak HR=0.026) in low and moderate-risk patients, respectively. Patients with high risk also continued to have two high mortality risk peaks, at 12 (peak HR=0.049) and 48 months (peak HR=0.055) (**Figure 7B**).

## Discussion

Our study revealed that IPI influence independently in long-term prognosis in ESCC patients after MIE and established a survival nomogram combining IPI and pathological features. When validated in an independent cohort, the nomogram showed better predictive performance and clinical utility than the traditional AJCC staging. We stratified the entire population using survival nomogram scoring criteria for OS and DFS and proposed SNS staging. Further analysis revealed that the risk of death varied depending on the SNS staging and changed over time.

Recent studies have proved that in addition to its tumor characteristics, the tumor microenvironment also promotes the proliferation and invasion of tumor cells (19, 20). The immune inflammatory reaction is an indispensable part of the tumor microenvironment, which is one of the mechanisms that promote tumor angiogenesis and immune resistance. Preoperative peripheral inflammatory markers can indirectly reflect the degree of immune inflammatory response in the tumor microenvironment and are promising prognostic indicators (4, 5). Mezquita et al. for the first time showed that the immune prognostic index (IPI), a new index of circulating inflammation, which was based on dNLR and LDH, could be an ideal prognostic tool for lung cancer (10). Past studies have confirmed that IPI is an independent prognostic factor in esophageal cancer (11, 21). Similarly, our results revealed that high preoperative IPI scores significantly reduced the OS and DFS in ESCC patients after minimally invasive surgery and were confirmed using an independent validation cohort.

In the present study, we found that age (> 65 years) and intraoperative bleeding (≥200 mL) were independent risk factors for adverse OS and DFS in ESCC patients. Older patients often present more comorbidities and poorer nutritional status than younger ones, and consequently higher mortality, as older patients are more likely to have serious complications and side effects after esophagectomy (22, 23). Although related studies have reported that blood transfusion can achieve short-term benefits in esophageal cancer patients with intraoperative massive bleeding, the immunosuppressive effect of allogeneic blood transfusion may reduce the antitumor activity of natural killer cells or T lymphocyte cells, resulting in poor prognosis in such patients (24, 25). Our study found no significant correlation between intraoperative blood loss and variables including age (**Supplementary Figure S6A**), adjuvant chemotherapy (**Supplementary Figure S6B**), grade T (**Supplementary Figure S6C**), grade N (**Supplementary Figure S6D**), tumor location (**Supplementary Figure S6E**), and surgical approach (**Supplementary Figure S6F**)-results consistent with previous studies (24). This may be attributed to the characteristics of the tumor types included in our study (e.g., esophageal squamous cell carcinoma), which primarily exhibit local infiltration and involve relatively fixed surgical scopes. Nevertheless, our multivariate Cox model, which included variables such as T stage and N stage, demonstrated that intraoperative blood loss remained independently associated with tumor characteristics (OS: HR=2.649, P=0.01; DFS: HR=2.450, P=0.01), indicating that its prognostic effect cannot be fully explained by tumor staging.

We observed significant correlations between intraoperative blood loss and ASA score, as well as postoperative complications (**Supplementary Figure S6G**). The ASA (American Society of Anesthesiologists) score is a classic indicator for assessing patients’ preoperative systemic status and directly reflects surgical risk (26). There was a significant positive correlation between intraoperative blood loss and ASA score (P=0.006), meaning patients with higher ASA scores (e.g., those with comorbidities such as cardiovascular or cerebrovascular diseases, or respiratory insufficiency) exhibited a significantly increased probability of higher intraoperative blood loss. A plausible mechanism for this phenomenon is that patients with higher ASA scores often present with coagulation dysfunction or impaired vascular regulation, making it more challenging to control intraoperative oozing during surgery. To reduce surgical risk, such patients may require more conservative surgical strategies (e.g., reduced resection extent), which indirectly increases the difficulty of intraoperative hemostasis and elevates blood loss.

More importantly, ASA score itself is an independent predictor of postoperative complications (e.g., infection, organ failure) (27). These complications directly worsen survival outcomes by prolonging hospitalization, increasing the risk of reoperation, or inducing multi-organ dysfunction. Thus, intraoperative blood loss may exert its prognostic effect through a “preoperative systemic status (ASA score) → intraoperative blood loss → postoperative complications → impaired survival” pathway, rather than merely serving as a surrogate for tumor characteristics. Beyond ASA score, we further identified a significant association between intraoperative blood loss and postoperative complications graded ≥ II using the Clavien-Dindo classification (CDc) (P=0.001) (**Supplementary Figure S6H**). Previous studies have demonstrated that postoperative complications not only pose a direct threat to short-term prognosis but also correlate with long-term survival. For instance, patients with anastomotic fistula exhibit a significantly lower 5-year overall survival rate than those without (HR=1.68, 95% CI: 1.25–2.24), which is closely linked to impaired tissue repair capacity and increased infection risk caused by blood loss (28). Therefore, intraoperative blood loss exacerbates survival disadvantages by directly inducing postoperative complications.

The traditional pathological tumor-node-metastasis (TNM) staging system has been widely used for risk stratification and prediction of survival. However, TNM staging only considers the characteristics of the tumor itself and does not include other prognostic factors, which limits its ability of survival prediction (29). Previous studies suggest that postoperative pathological staging combined with other clinical prognostic factors (age, postoperative adjuvant therapy, tissue differentiation, lymphatic vascular invasion, etc.) can predict the long-term oncology outcomes more accurately (30–32). Feng et al. established a nomogram including IPI score to predict cancer-specific survival in ESCC patients, but the sample size included in this study was small, and most of the study population underwent OE. Moreover, the nomogram developed in the study was not validated in other independent cohorts (11). In the present study, we established nomograms that could visually predict OS and DFS in ESCC patients after MIE by considering both IPI scores as well as clinicopathological features. The nomogram obtained in this way had better predictive power than TNM staging by t-AUC curves. In addition, the DCA showed that using this nomogram, the rate of accuracy to predict OS or DFS was consistently higher than that of TNM staging over a wide range of thresholds.

Previous studies on constructing nomogram models for ESCC have largely relied on static anatomical or pathological variables, which fail to comprehensively reflect the dynamic characteristics of tumor biological behavior. For instance, Li et al. (2022) incorporated preoperative/postoperative inflammatory indicators (PreSII, PostSII) and a nutritional index (PNI) but did not address dynamic changes in the immune microenvironment (33). Zhang et al. (2021) focused on anatomical features such as tumor length and lymphovascular space invasion (LVSI), showing significant deficiencies in assessing systemic immune status (34). Deng et al. (2019) only integrated traditional clinicopathological factors (e.g., TNM staging, age), resulting in a single-dimensional predictive model that could not dissect the core drivers of ESCC heterogeneity (32).

Innovatively, our study incorporated the immune prognostic index (IPI, calculated as dNLR + LDH) into the predictive framework. By quantifying the synergistic effects of systemic inflammatory burden (dNLR) and tumor metabolic activity (LDH), we dynamically evaluated the perioperative immune microenvironment status. Additionally, we included intraoperative blood loss (≥200 mL)—a key indicator reflecting surgical stress—thereby filling the knowledge gap in traditional models regarding immune-metabolic crosstalk and perioperative impacts.

In terms of predictive performance, the survival nomogram scoring system (SNS) developed in this study demonstrated significant advantages. The time-dependent area under the curve (AUC) for 5-year overall survival (OS) reached 0.780 in the training cohort and increased to 0.827 in the validation cohort. This performance not only substantially outperformed the AJCC 8th edition staging system (training/validation AUC: 0.687/0.750) but also surpassed the traditional pathological model reported by Deng et al. (training/validation AUC: 0.685/0.744) (32). Notably, although the AUC values of models by Li et al. (training/validation: 0.850/0.797) and Zhang et al. (training/validation: 0.802/0.829) were comparable to ours, our study was based on a large-sample ESCC cohort (613 patients; training set: n=425, validation set: n=188), with a significantly larger sample size than those of Li et al. (n=268) and Zhang et al. (n=399) (33, 34). This provided a more robust statistical foundation and stronger generalizability for our model.

To make nomograms practical in clinical application, we established threshold values for risk assessment. In the training cohort, we developed accurate prediction scores for OS (low risk: ≤170; moderate risk:170 - 219; high risk: >219) and DFS (low risk: ≤167; moderate risk: 167 - 227; high risk: >227). The survival prediction ability of SNS staging was also confirmed in the validation cohort. Further exploration of the dynamic mortality risk in different SNS stagings revealed that the risk of death in patients with high probability showed a double peak, at 12 and 48 months after surgery, respectively. Patients with moderate risk had the highest probability of death at 12 months after surgery, while patients with low risk had the greatest probability of death at 24 months after surgery, and then the risk of death decreased. This finding provides precise temporal anchors for clinical decision-making, such as enhancing imaging surveillance and implementing intervention measures at 12 and 48 months for high-risk groups.

The present study, for the first time, combined immunoinflammatory indicators and clinicopathological features to establish a new comprehensive staging system, SNS staging, which can effectively predict the prognosis in ESCC patients after MIE. However, there are some limitations to our study. First, the selection and analysis biases are inevitable because of single-center retrospective analysis, and more external data is required for validation. Although referencing the dNLR and LDH cutoff values from the gastric cancer cohort allowed us to preliminarily evaluate the prognostic value of the IPI in ESCC (12), the extrapolation validity of these cutoff values requires cautious interpretation. In the future, it will be necessary to re-optimize the thresholds in larger, multicenter ESCC cohorts using internal statistical methods (e.g., ROC curve analysis to determine optimal cutoff values and the X-tile method to dynamically stratify risk groups) and further enhance the generalizability and clinical utility of the IPI score by integrating clinical validation (e.g., consistency of survival curves and relevance to clinical decision-making).Second, our nomogram and comprehensive SNS staging were based on the data of ESCC patients undergoing only surgery, and their clinical significance needs to be further confirmed for patients undergoing neoadjuvant plus surgery. Finally, the IPI score in this study was measured at a single time point before surgery. The impact of postoperative IPI on the prognosis in ESCC patients and the dynamic changes in IPI should be assessed further.

Overall, the survival nomogram combining IPI and clinicopathological features gives a comprehensive prediction model by considering tumor characteristics and immune-inflammatory responses, which can accurately evaluate the long-term outcomes in ESCC patients after minimally invasive surgery. The predictive performance and clinical benefit rate of the survival nomogram were higher than those in conventional AJCC staging. More importantly, based on the survival nomogram score, we developed SNS staging for OS and DFS respectively, which can provide reference values in clinical follow-up.

## Data Availability

The raw data supporting the conclusions of this article will be made available by the authors, without undue reservation.

## Supplementary material

Supplementary Figure 1 Kaplan Meier survival curve of ESCC patients in training cohort. **(A)** OS and **(B)** DFS for dNLR. **(C)** OS and **(D)** DFS for LDH.

Supplementary Figure 2 Kaplan Meier survival curve of ESCC patients in validation cohort. **(A)** OS and **(B)** DFS for dNLR. **(C)** OS and **(D)** DFS for LDH.

Supplementary Figure 3 ROC curve reveals the ability of dNLR, LDH and IPI to predict 5-year OS and 5-year DFS in (**A** and **B**) training cohort and in (**C** and **D**) validation cohort.

Supplementary Figure 4 Calibration curves of nomogram for OS (**A**, **C** and **E**) and DFS (**B**, **D** and **F**) in training cohort.

Supplementary Figure 5 Calibration curves of nomogram for OS (**A**, **C** and **E**) and DFS (**B**, **D** and **F**) in validation cohort.

Supplementary Figure 6 Relationship between intraoperative blood loss and patient age, adjuvant chemotherapy, T stage, N stage, tumor location, surgical approach, ASA score and postoperative complications.
