# Supplementary figures and images for: Establishment and validation of survival nomogram score staging for esophageal squamous cell carcinoma patients after minimally invasive surgery combined with immune prognostic index and clinicopathological features
Source: Front Immunol. 2025 Sep 17;16:1635000. doi: 10.3389/fimmu.2025.1635000 (PMC12484022; doi:10.3389/fimmu.2025.1635000)

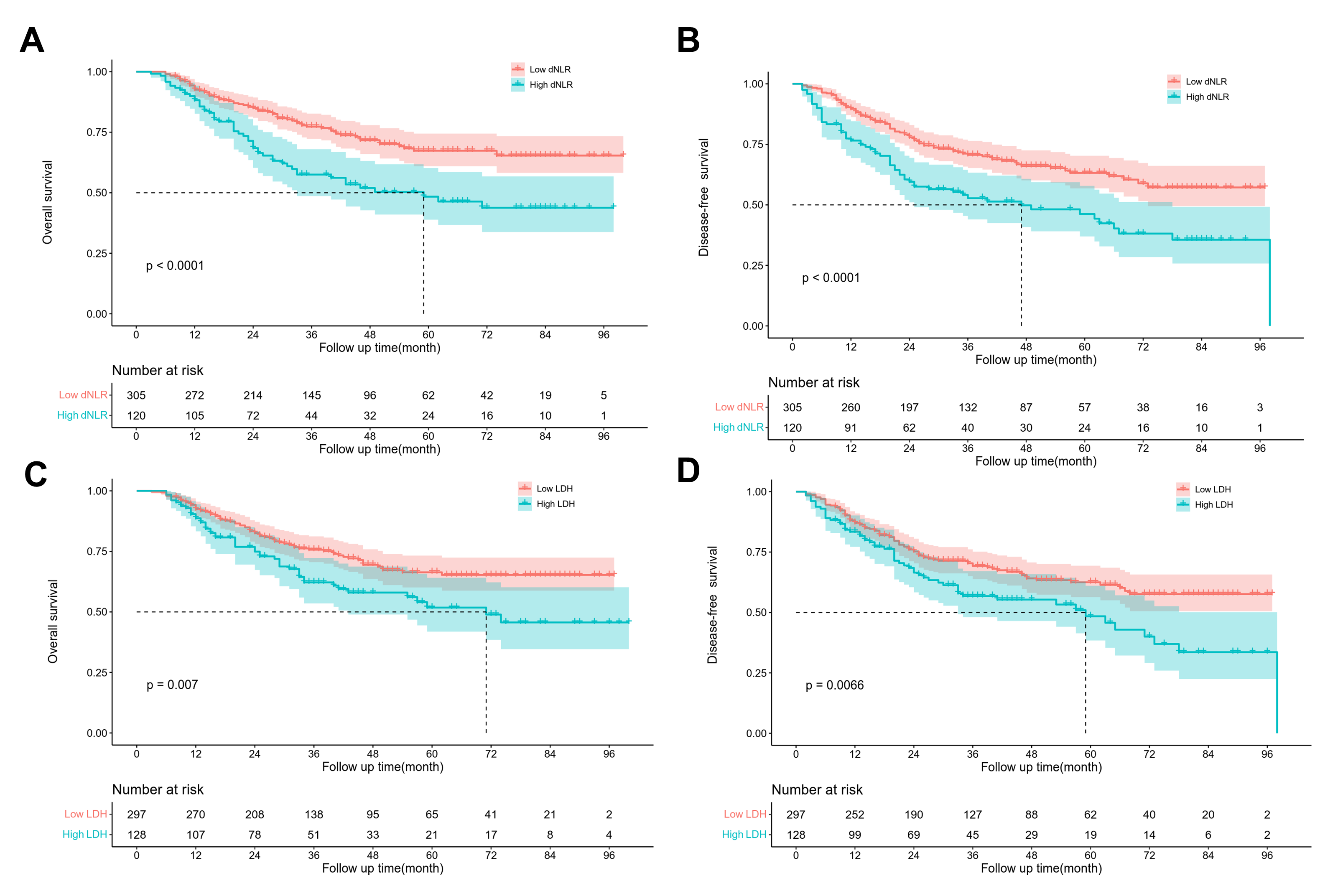

Supplement: Supplementary Figure 1 — Kaplan Meier survival curve of ESCC patients in training cohort. (A) OS and (B) DFS for dNLR. (C) OS and (D) DFS for LDH. [file Image1.tif]

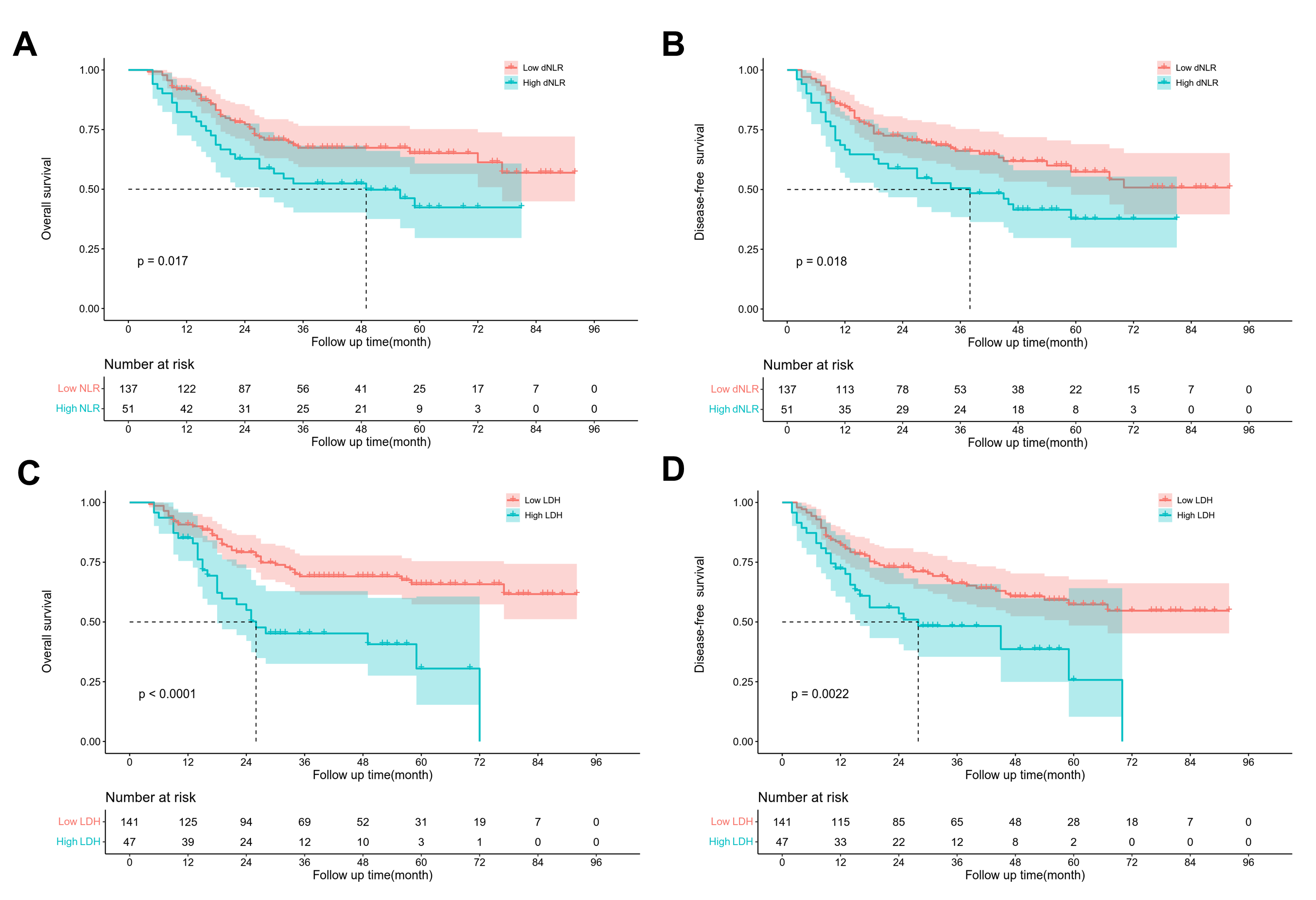

Supplement: Supplementary Figure 2 — Kaplan Meier survival curve of ESCC patients in validation cohort. (A) OS and (B) DFS for dNLR. (C) OS and (D) DFS for LDH. [file Image2.tif]

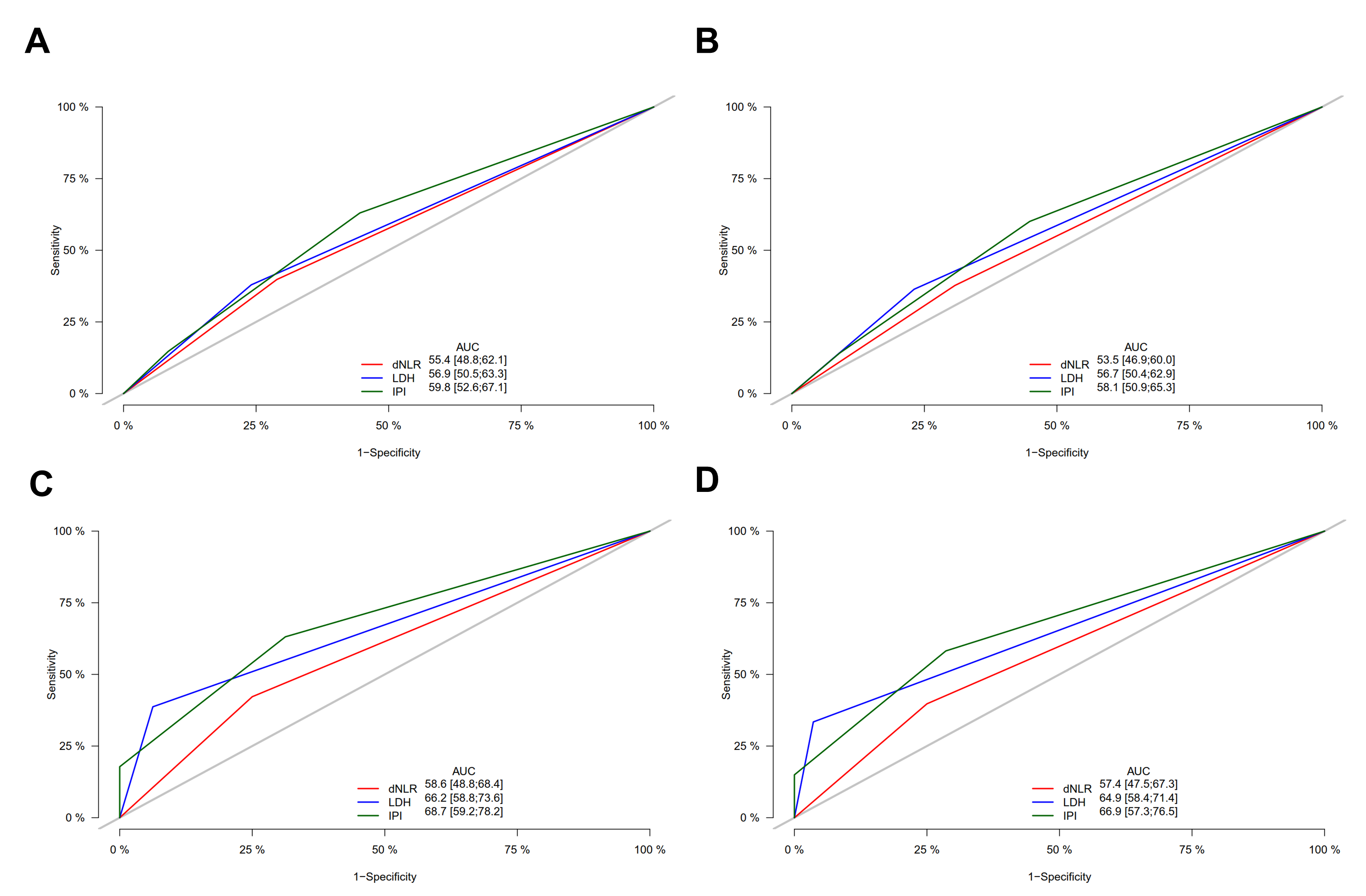

Supplement: Supplementary Figure 3 — ROC curve reveals the ability of dNLR, LDH and IPI to predict 5-year OS and 5-year DFS in (A and B) training cohort and in (C and D) validation cohort. [file Image3.tif]

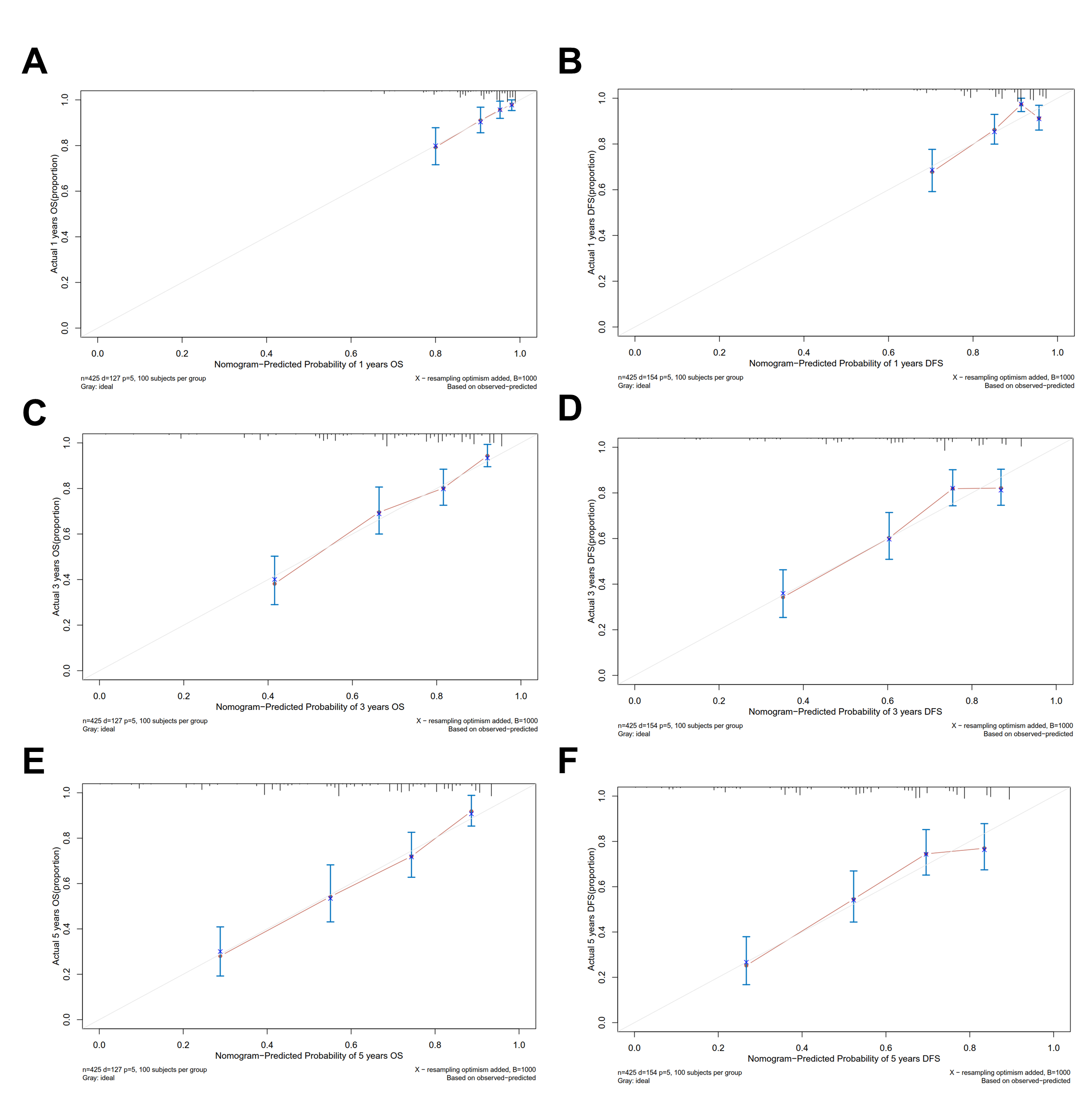

Supplement: Supplementary Figure 4 — Calibration curves of nomogram for OS (A, C and E) and DFS (B, D and F) in training cohort. [file Image4.tif]

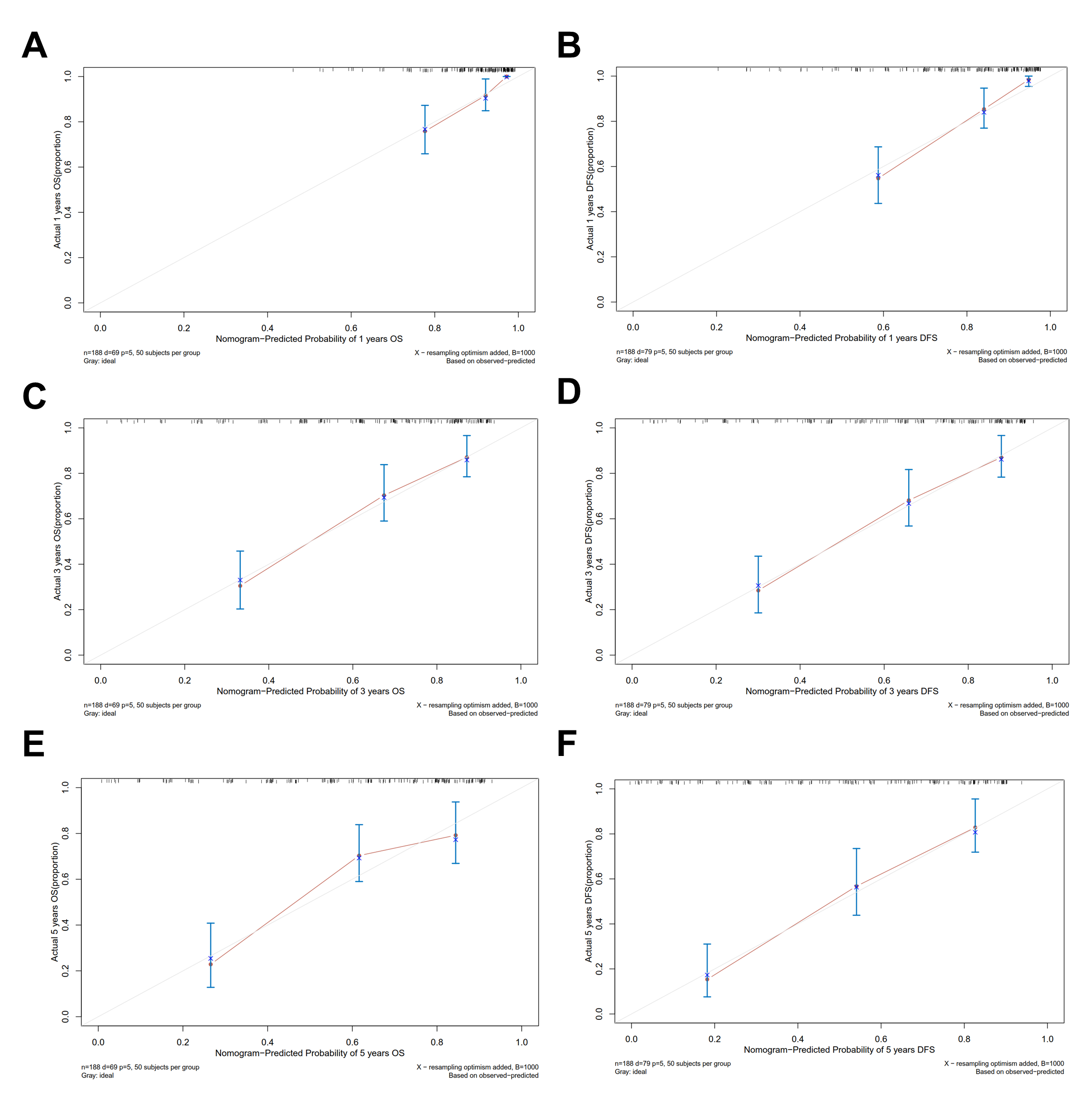

Supplement: Supplementary Figure 5 — Calibration curves of nomogram for OS (A, C and E) and DFS (B, D and F) in validation cohort. [file Image5.tif]

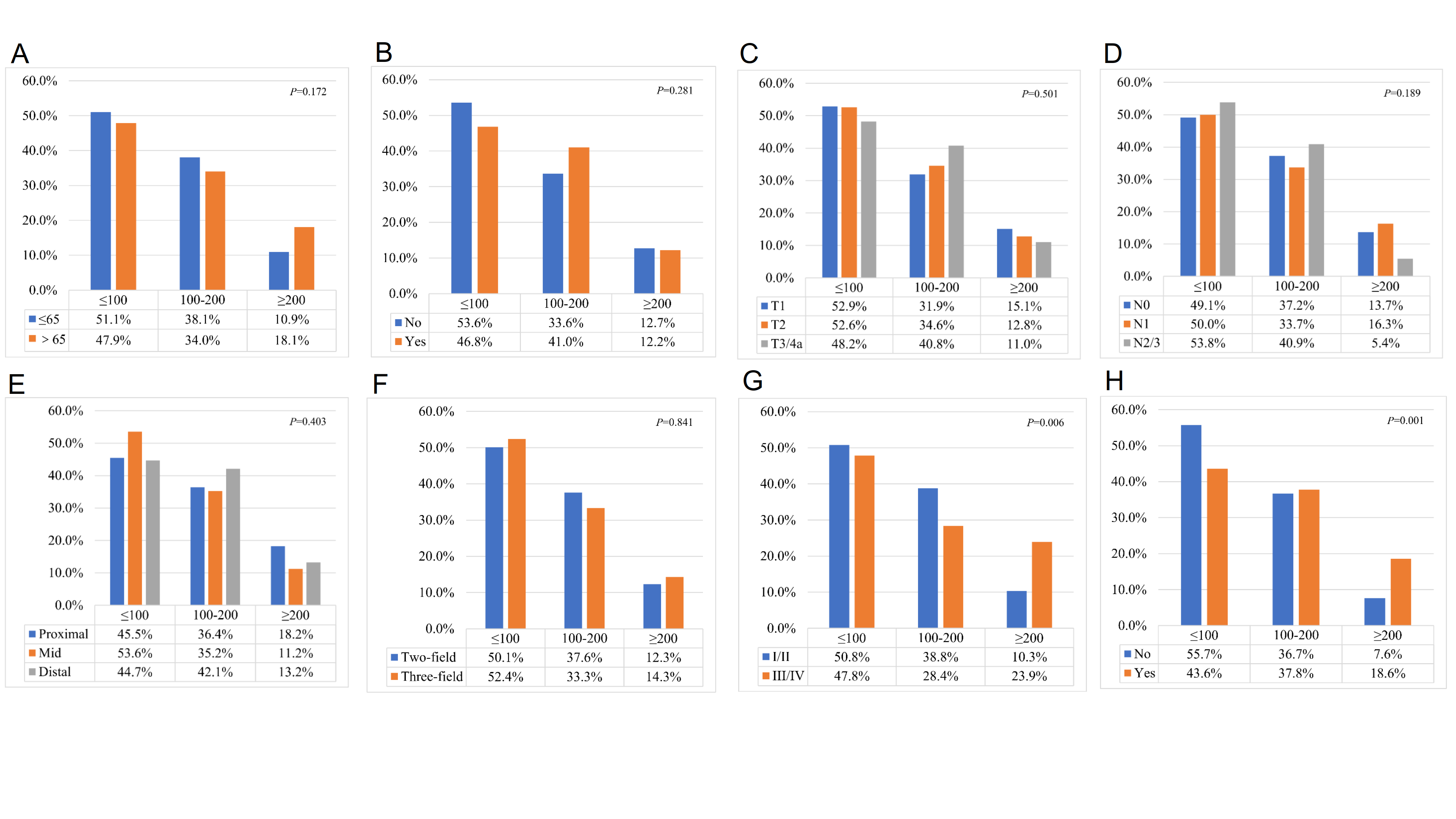

Supplement: Supplementary Figure 6 — Relationship between intraoperative blood loss and patient age, adjuvant chemotherapy, T stage, N stage, tumor location, surgical approach, ASA score and postoperative complications. [file Image6.tif]
